# Comparison of the 2010 and 2019 diagnostic criteria for sarcopenia by the European Working Group on Sarcopenia in Older People (EWGSOP) in two cohorts of Swedish older adults

**DOI:** 10.1186/s12877-021-02533-y

**Published:** 2021-10-26

**Authors:** Ola Wallengren, Ingvar Bosaeus, Kerstin Frändin, Lauren Lissner, Hanna Falk Erhag, Hanna Wetterberg, Therese Rydberg Sterner, Lina Rydén, Elisabet Rothenberg, Ingmar Skoog

**Affiliations:** 1 Department of Internal Medicine and Clinical Nutrition, Institute of Medicine, Sahlgrenska Academy, University of Gothenburg, Gothenburg, Sweden; 2 Clinical Nutrition Unit, Sahlgrenska University hospital, Gothenburg, Sweden; 3 Department of Psychiatry and Neurochemistry, Institute of Neuroscience and Physiology, Sahlgrenska Academy, Centre for Ageing and Health (AgeCap) at the University of Gothenburg, Gothenburg, Sweden; 4 School of Public Health and Community Medicine, Institute of Medicine, Sahlgrenska Academy, University of Gothenburg, Gothenburg, Sweden; 5 Region Västra Götaland, Sahlgrenska University Hospital, Psychiatry, Cognition and Old Age Psychiatry Clinic, Gothenburg, Sweden; 6 Faculty of Health Sciences, Kristianstad University, Kristianstad, Sweden

**Keywords:** Diagnostic criteria, Sarcopenia, Mortality, EWGSOP, Muscle mass, Gait speed, Handgrip strength, Population sample, Ageing, Activities of daily living, H70

## Abstract

**Background:**

The operational definition of sarcopenia has been updated (EWGSOP2) and apply different cut-off points compared to previous criteria (EWGSOP1). Therefore, we aim to compare the sarcopenia prevalence and the association with mortality and dependence in activities of daily living using the 2010 (EWGSOP1 and 2019 (EWGSOP2 operational definition, applying cut-offs at two levels using T-scores.

**Methods:**

Two birth cohorts, 70 and 85-years-old (*n* = 884 and *n* = 157, respectively), were assessed cross-sectionally (57% women). Low grip strength, low muscle mass and slow gait speed were defined below − 2.0 and − 2.5 SD from a young reference population (T-score). Muscle mass was defined as appendicular lean soft tissue index by DXA. The EWGSOP1 and EWGSOP2 were applied and compared with McNemar tests and Cohen’s kappa. All-cause mortality was analyzed with the Cox-proportional hazard model.

**Results:**

Sarcopenia prevalence was 1.4–7.8% in 70-year-olds and 42–62% in 85 years-old’s, depending on diagnostic criteria. Overall, the prevalence of sarcopenia was 0.9–1.0 percentage points lower using the EWGSOP2 compared to EWGSOP1 when applying uniform T-score cut-offs (*P* <  0.005). The prevalence was doubled (15.0 vs. 7.5%) using the − 2.0 vs. -2.5 T-scores with EWGSOP2 in the whole sample. The increase in prevalence when changing the cut-offs was 5.7% (*P* <  0.001) in the 70-year-olds and 17.8% (*P* <  0.001) in the 85-year-olds (EWGSP2). Sarcopenia with cut-offs at − 2.5 T-score was associated with increased mortality (hazard ratio 2.4–2.8, *P* <  0.05) but not at T-score − 2.0.

**Conclusions:**

The prevalence of sarcopenia was higher in 85-year-olds compared to 70-year-olds. Overall, the differences between the EWGSOP1 and EWGSOP2 classifications are small. Meaningful differences between EWGSOP1 and 2 in the 85-year-olds could not be ruled out. Prevalence was more dependent on cut-offs than on the operational definition.

**Supplementary Information:**

The online version contains supplementary material available at 10.1186/s12877-021-02533-y.

## Introduction

According to the European Working Group on Sarcopenia in Older People (EWGSOP), sarcopenia can be defined as the combination of low muscle mass and poor muscle function [1, 2]. The concept was originally introduced by Rosenberg in 1988 [3] and is a geriatric syndrome associated with adverse effects on function, quality of life, and survival [1, 4, 5]. Various definitions have subsequently been proposed [2, 5, 6]. In 2016, the World Health Organization (WHO) launched the International Classification of Disease (ICD) code for sarcopenia [7]. Recently, the EWGSOP published an updated consensus definition that uses poor muscle strength as the key characteristic for the condition rather than low muscle mass (EWGSOP2) [2]. The EWGSOP2 excludes slow gait speed as a diagnostic criterion for sarcopenia, resulting in a lower prevalence [8–10]. Several studies comparing EWGSOP1 and 2 have been published. Some studies show good agreement [9, 11–13], but others find poor agreement between the two criteria [8, 10, 14–21]. Populations, settings, techniques, testing procedures, and cut-offs have varied, leading to differences in the prevalence of sarcopenia, making comparisons between studies difficult [8, 11–22]. Few studies have compared the EWGSOP1 and 2 in a representative and age-standardized sample of community-dwelling older adults [11, 21], and so far, only two (Asian) studies has applied regional cut-offs and also compared with cut-offs suggested by the EWGSOP [9, 10]. There is a lack of knowledge about how EWGSOP1 and 2 impact the prevalence of sarcopenia that is distinct from applying different cut-offs. The objective of this study was, therefore to cross-sectionally compare the difference of the EWGSOP1 and 2 operational definitions on sarcopenia prevalence, applying cut-offs based on T-scores at two levels (− 2.0 and − 2.5) in two population-based samples of 70 and 85-year-olds from Gothenburg, Sweden.

## Methods

### Study population

This study is part of the Gothenburg H70 Birth Cohort Studies in Sweden (H70 studies) [23]. The studies are multidisciplinary population studies examining systematically recruited birth-cohorts of older populations in Gothenburg, Sweden, via the Swedish Population Register (Statistics Sweden).

The present study includes one cohort born in 1944, examinations started in 2014 at age 70 (*n* = 1203 response rate 72%), and one cohort born in 1930, examinations started in 2015 at age 85 (*n* = 491, response rate 64%). In this 1930-cohort, 75 individuals lived in Gothenburg at inclusion in a previous examination in the H70 study but have moved from the city before the present examination. The study has a cross-sectional design except for survival that was checked on June 25, 2020 (Swedish population register). Follow-up time ranged from 2.9 to 6.3 years. The birth cohorts and characteristics of participants and non-participants have been described in detail previously [23–25]. All subjects gave informed consent, and the Regional Ethical Review Board in Gothenburg approved the studies.

### Participant characteristics

Participants provided information regarding smoking habits, medications, cohabitation, and education level during a semi-structured face-to-face interview. Self-rated health was assessed using the general question; how would you rate your health?”, with response options ranging from very good to very poor. Leisure-time physical activity was classified on four levels with a validated question: sedentary (mainly reading or watching television), moderate (walking outdoors or cycling regularly), regular (sports or strenuous gardening ≥3 h per week), and athletic (regular strenuous physical activity) [26]. Independence in activities of daily living (ADL) was assessed with the Barthel index [27], which includes ten domains of function (bowels, bladder, grooming, toilet use, feeding, transfer, mobility, dressing, stairs, and bathing). Response options are either independent or dependent/unable and summed to a total score ranging from zero (low function, dependent) to 100 (high function, independent). A score <  100 indicates ADL dependence.

### Anthropometry

Bodyweight was measured with a calibrated electronic medical scale and body height with a stadiometer. Mid-arm circumference was measured on the right arm, relaxed mid-humerus, with a measuring tape. Body composition was measured by Dual-energy X-ray absorptiometry (DXA) using a Lunar Prodigy scanner in the 70-year-olds and Lunar iDXA (GE Health Care) in the 85-year-olds. Whole-body scans were performed, and lean soft tissue was analyzed (enCORE software version 12.30.008 and 14.10.022, respectively). Appendicular lean soft tissue index (ALSTI, kg/m^2^), defined as the sum of lean soft tissue in arms and legs divided by body height squared, was used as an estimate of muscle mass. Scanners were cross calibrated by a double scan of 33 subjects (12 men, 21 women) on the same day. iDXA ALST (appendicular lean soft tissue) measurements were calibrated with Prodigy using a regression equation with ALST, fat mass, and sex as predictors (*R*^*2*^ = 0.992, RMSE = 0.55 kg).

### Strength and performance

Handgrip strength was measured with a Martin Vigorimeter (Gebrüder Martin GmbH & Co, Tuttlingen, Germany) with the shoulder joint in a neutral position. The large bulb was used for men and the medium bulb for women. The test was repeated three times for each hand, and the highest value of the strongest hand was used. Self-selected gait speed was measured over 30-m indoors with standing start (meter/second).

### Sarcopenia definitions

Sarcopenia was defined using both the 2010 and the 2019 operational definitions proposed by the EWGSOP, Table 1 [1, 2]. When low muscle strength, low muscle quantity, and low physical performance are all detected, sarcopenia is considered severe [1, 2]. T-score based cut-offs were applied at two levels, − 2.0 and − 2.5 T-score, based on a regional normative reference population, Table 3. Cut-offs as originally published by the EWGSOP 1 and 2 were also applied for comparison (here termed “original”), except for hand grip strength were a cut-off of − 2.5 T-scores were applied, since no cut-offs for the Martin Vigorimeter have been published by the EGWSOP, Table 3 [1, 2]. In this way we studied the impact of the EWGSOP 1 and 2 operational definitions, with the same cut-offs, and the impact of different cut-off points on the prevalence of sarcopenia. T-scores for hand grip strength were estimated from healthy subjects aged 20–45 years (125 men, 125 women) from Sweden using the Martin Vigorimeter [28] (Table 3). T-scores for gait speed was estimated by a meta-analysis (pooling) of studies of healthy adults (30–59 years) from Sweden (Gothenburg area), measured over 30 m in “normal” walking speed with a standing start (67 men, 68 women) [29, 30]. Low muscle mass was defined as ALSTI measured by DXA below a T-score of − 2.0 and − 2.5 in Swedish healthy adults (Gothenburg area) aged 20–40 years (183 men, 239 women) [31] (Table 3).

**Table 1:** Operational definition of sarcopenia and severe sarcopenia according to EWGSOP1 and 2

Sarcopenia criteria	Low muscle mass	Low muscle strength	Low physical performance
EWGSOP1	✓	✓ Or ✓	
EWGSOP2	✓	✓	
EWGSOP1 & 2, severe	✓	✓	✓

EWGSOP; European Working Groupon Sarcopenia in Older People 2010 (EWGSOP1) [1] and 2019 (EWGSOP2) [2]

### Statistics

Data are presented as group mean ± standard deviation (SD) and proportions and prevalences as count and percent (%). Differences in means were tested with Student’s t-test and differences in proportions with Pearson’s Chi2-test. Differences in paired proportions (i.e. Sarcopenia classifications) were tested with McNemar’s exact test (binomial test) with confidence intervalls calculated according to Newcombe [32] and expressed as difference in percentge points. Cohen’s kappa were used to show agreement between classifications, rated according to Landis and Koch [33]. Differences in mortality between the cohorts and between participants vs. non-participants were tested with log-rank test. Associations with sarcopenia classifications and mortality were tested with Cox proportional-hazards model and associations with ADL dependence (Barthel index< 100) were tested with logistic regression. Models were adjusted for age (cohort) and sex. Result are presented as Hazard-ratios (HR) and Odds-ratios (OR) with 95% confidence interval. Statistical analyses were performed IBM SPSS statistics 26.0 software program (IBM Corp., Armonk, NY). The tests were considered significant at the level of *P* <  0.05 in two-tailed analyses.

## Result

### Population characteristics

Data on all diagnostic criteria measures were available for 1041 participants, 884 70-year old’s and 157 85-year-olds (i.e., 73 and 32% of those participating in the general exams, respectively). Characteristics of the samples are presented in Table 2.

**Table 2:** Subject characteristics

	Cohort		
	70-year-olds *n* = 884	85-year-olds *n* = 157	Cohort Difference
	Mean ± SD	Mean ± SD	*P*-value^a^
Age (years)	70.5 ± 0.3	86.0 ± 0.2	–
Sex men/women, n (women %)	391/493 (56%)	57/100 (64%)	0.07
BMI (kg/m^2^)	26.2 ± 4.3	25.8 ± 4.0	0.39
BMI class (%)			
< 18.5	1	0	0.51
18.5–25	43	44	
25–30	40	41	
> 30	16	15	
Appendicular lean soft tissue (kg/m^2^)			
Men	7.9 ± 0.8	6.8 ± 0.8	< 0.001
Women	6.2 ± 0.6	5.4 ± 0.6	< 0.001
Handgrip strength (kPa)			
Men	87 ± 15	58 ± 11	< 0.001
Women	74 ± 14	49 ± 14	< 0.001
Gait speed 30 m. (m/s)			
Men	1.3 ± 0.2	1.1 ± 0.2	< 0.001
Women	1.3 ± 0.2	1.0 ± 0.2	< 0.001
Arm circumference (cm)			
Men	30.2 ± 3.3	28.8 ± 3.4	0.004
Women	28.8 ± 3.5	27.7 ± 3.6	0.003
Medications (n)	4.0 ± 3.4	5.6 ± 3.9	< 0.001
Smoker (%)			
Never	41	48	0.04
Former	52	50	
Current	7	3	
Physical activity (%)			
Sedentary	2	6	< 0.001
Light to moderate	11	34	
Active	49	53	
Athletic	38	7	
Self-rated health (%)			
Very good	38	24	0.007
Fair to good	55	64	
Decent to bad	7	11	
Very bad	0	0	
Education (%)			
Primary	12	45	< 0.001
Secondary or more	88	55	
Barthel index < 100 (%)			
Need help	14	25	0.002
Living alone (%)	36	64	< 0.001
Deceased, n (%)	29 (3)	24 (15)	< 0.001^b^

^a^Difference between cohorts, T-test or Chi^2^

^b^Log-rank test

There was a higher proportion who died during the 3 to 6 years of follow-up among non-participants, i.e., in those not taking part in the sarcopenia study compared to the participants, in both cohorts. Among men in both cohorts, and 70-year-old women, there were a higher proportion of smokers among non-participants. Among 70-year-old women there was a higher proportion with poor self-rated health and the same tendency was also shown among 85-year-old women, however not significant. In the 70-year-olds, participants in the sarcopenia study had higher education, lower BMI and faster gait speed compared to non-participants. In the 85-year-olds, non-participants used more medications than participants, see Table S1, Additional file 1.

### Sarcopenia prevalence

The prevalence of reduced hand grip strength, gait speed and muscle mass at the T-score cut-offs (T-scores − 2.0 and − 2.5) and at the originally published cut-offs are shown in Table 3. The prevalence of reduced hand grip strength, gait speed and muscle mass were higher among the 85-year-olds compared to the 70-year-olds, both among men and women (*P* <  0.001).

**Table 3:** Prevalence (%) of reduced hand grip strength, gait speed and muscle mass

Measure	Cut-offs^a^			Prevalence (%)			
				70-year-olds		85-year-olds	
	Criteria	Men	Women	Men	Women	Men	Women
Grip strength, (kPa)	T-score − 2.0	< 73	< 68	18	35	89	91
	T-score − 2.5	< 69	< 59	10	12	81	75
Gait speed 30 m, (m/s)	T-score − 2.0	< 1.09	< 1.08	9.2	10	49	59
	T-score − 2.5	< 1.00	< 0.98	5.1	3.4	33	40
	EWGSOP1&2	< 0.80	< 0.80	1.5	0.6	8.8	12
Muscle Mass, (ALSTI, kg/m^2^)^b^	T-score − 2.0	< 6.92	< 5.65	11	20	56	68
	T-score − 2.5	< 6.53	< 5.45	3.8	10	37	59
	EWGSOP1	< 7.26	< 5.45	21	10	74	59
	EWGSOP2	< 7.00	< 5.50	13	12	63	64

^a^ Cut-offs according to European Working Groupon Sarcopenia in Older People 2010 (EWGSOP1) [1], 2019 (EWGSOP2) [2] and population specific T-scores (− 2.0 and − 2.5)

^b^ Appendicular lean soft tissue index; ALSTI, measured by Dual-energy x-ray absorptiometry

The prevalence of sarcopenia and severe sarcopenia was higher in 85-year-olds compared to 70-year-olds irrespective of diagnostic criteria (*P* < 0.001) (Table 4). There was sex difference in the prevalence of sarcopenia among 70-year-olds when applying the − 2.0 T-score cut-offs for EWGSOP1 and 2 and the EWGSOP 1 with original cut-offs (Table 4). Among the 85-year-olds there was a sex difference when applying the EWGSOP1 T-score − 2.5 (Table 4). The prevalence of low hand grip strength without sarcopenia (EWGSOP2 T-score − 2.5) were 10 and 35% in the 70 and 85-year-olds, respectively. Consequently, 13 and 55% of those who had low hand grip strength (termed probable sarcopenia according to EWGSOP2) in the 70 and 85-year-olds respectively were confirmed sarcopenic according to EWGSOP2 (T-score − 2.5). There were no cases of severe sarcopenia in the 70-year-olds using the original EWGSOP 1 or 2 criteria.

**Table 4:** Sarcopenia prevalence (%) according to EWGSOP1 and 2 at selected cut-offs

Sarcopenia criteria	Sarcopenia prevalence (%)						
EWGSOP (cut-offs)^a^	70-year-olds			85-year-olds			
	Men	Women	Both sexes	Men	Women	Both sexes	All
EWGSOP 1 (T-score − 2.0)	5.1	9.9^b^	7.8	56	65	62	16
EWGSOP 2 (T-score − 2.0)	3.8	9.5^b^	7.0	54	63	60	15
EWGSOP 1 (T-score − 2.5)	1.8	2.0	1.9	33	51^b^	45	8.4
EWGSOP 2 (T-score − 2.5)	1.0	1.6	1.4	33	47	42	7.5
EWGSOP 1 (original)^a^	4.6	1.8^b^	3.1	61	50	54	11
EWGSOP 2 (original)^a^	3.6	2.2	2.8	53	54	54	10
EWGSOP 1 or 2, severe (T-score − 2.0)	0.5	1.8	1.2	25	35	31	5.8
EWGSOP 1 or 2, severe (T-score − 2.5)	0.3	0.2	0.2	12	23	19	3.1
EWGSOP 1, severe (original)^a^	0	0	0	3.5	6.0	5.1	0.8
EWGSOP 2, severe (original)^a^	0	0	0	3.5	6.0	5.1	0.8

EWGSOP; The European Working Groupon Sarcopenia in Older People 2010 (EWGSOP1) [1] and 2019 (EWGSOP2) [2] diagnostic criteria (Table 1 and Table 3)

^a^ Cut-offs as published by the EWGSOP 1 and 2 (original) [1, 2] and population based T-scores (−2.0 and − 2.5) (Table 3)

^b^ Difference between sexes within age cohort (*P* < 0.05)

Agreement between the EWGSOP 1 and 2 was “almost perfect” (Cohen’s kappa, range 0.82–1.00), except for 70-year-old men when applying the − 2.5 T-scores (Table 5).

**Table 5:** Difference in sarcopenia prevalence (percentage points, %) between EWGSOP1 and 2 applying uniform cut-offs (T-score − 2.0 and − 2.5)

	EWGSOP (cut-offs)			EWGSOP (cut-offs)		
	1 vs. 2 (T-score − 2.0)			1 vs. 2 (T-score − 2.5)		
Cohort/Sex (n)	Difference % (95%CI)	*P*-value^a^	*Κ*^b^	Difference % (95%CI)	*P*-value^a^	*Κ*^b^
All (1041)	1.0 (0.3–1.7)	0.002	0.96	0.9 (0.2–1.6)	0.004	0.94
70-yrs						
Both sexes (884)	0.8 (0.1–1.6)	0.016	0.94	0.6 (0.0–1.3)	0.063	0.82
Men (391)	1.3 (−0.1–2.9)	0.063	0.85	0.8 (−3.6–2.3)	0.25	0.72
Women (493)	0.4 (−0.5–1.4)	0.50	0.98	0.4 (−0.5–1.5)	0.50	0.89
85-yrs						
Both sexes (157)	1.9 (−0.6–4.4)	0.25	0.96	2.5 (−0.2–5.3)	0.13	0.95
Men (57)	1.8 (− 3.0–6.5)	1.0	0.96	0.0 (− 3.9–3.9)	1.0	1.0
Women (100)	2.0 (−1.4–5.5)	0.50	0.96	4.0 (−0.3–8.2)	0.13	0.92

EWGSOP; The European Working Groupon Sarcopenia in Older People 2010 (EWGSOP1) [1] and 2019 (EWGSOP2) [2] diagnostic criteria (Table 1 and Table 3)

^a^ Difference, Exact binomial sign test

^b^ Agreement, Cohen’s kappa

Overall, the prevalence of sarcopenia was 1.0 and 0.9 percentage points lower using the EWGSOP 2 compared to EWGSOP 1 when applying the T-score − 2.0 cut-offs (15.0 vs. 15.9%, *P* = 0.008) and − 2.5 cut-offs (7.5 vs. 8.4%, *P* = 0.008) respectively (Table 5). There were significant differences in prevalence using the EWGSOP 2 compared to EWGSOP 1 with T-score − 2.0 in the 70-year-olds. No significant differences were found in the 85-year-olds, but confidence intervals of the difference were wide, including a difference of 3.9 to 8.2% (Table 5).

Agreement between the EWGSOP 1 and 2 when applying original cut-offs was “almost perfect” (Cohen’s kappa, range 0.82–0.92) (Table S2, Additional file 1). There was no significant difference in prevalence between EWGSOP 2 and 1 (Δ -0.3%, 10.5 vs. 10.8%, *P* = 0. 61) with original cut-offs. Among the 85-year-old men this difference nearly reached significance (Δ -8.8%, 52.6 vs. 61.4%, *P* = 0.063) (Table S2, Additional file 1).

There were significant differences in sarcopenia prevalence applying the − 2.0 vs. -2.5 T-score cut-offs to the EWGSOP1 and 2 operational definitions (*P* < 0.001) (Table 6). Overall, the prevalence was doubled (15.0 vs. 7.5%) using the − 2.0 vs. -2.5 T-scores with EWGSOP2. The increase in prevalence when changing the cut-offs was relatively larger in the 70-year-olds from 1.4 to 7.0% (5-fold) than in the 85-year-olds but were larger in absolute terms in the older cohort (Δ + 17.8%, 42 to 60%, *P* < 0.001) (Tables 4 and 6). The prevalence of severe sarcopenia increased from 0.2 to 1.2% (*P* = 0.004) in the 70-year-olds and from 19 to 31% (Δ + 12.1%, *P* < 0.001) in the 85-year-olds when changing cut-offs from T-score − 2.5 to − 2 (see Table S3, Additional file 1**)**.

**Table 6:** Difference in sarcopenia prevalence at T-score cut-offs − 2.0 vs. -2.5 using the EWGSOP1 and 2 operational definitions

	Sarcopenia (cut-offs)			Sarcopenia (cut-offs)		
	EWGSOP1 T-score − 2.0 vs. -2.5			EWGSOP2 T-score − 2.0 vs. -2.5		
Cohort/Sex (n)	Difference % (95%CI)	*P*-value^a^	*Κ*^b^	Difference % (95%CI)	*P*-value^a^	*Κ*^b^
All (1041)	7.6 (6.0–9.3)	< 0.001	0.65	7.5 (5.9–9.2)	< 0.001	0.63
70-yrs						
Both sexes (884)	5.9 (4.4–7.6)	< 0.001	0.38	5.7 (4.2–7.4)	< 0.001	0.31
Men (391)	3.3 (1.6–5.6)	< 0.001	0.51	2.8 (1.2–5.0)	< 0.001	0.41
Women (493)	7.9 (5.7–10.6)	< 0.001	0.32	7.9 (5.7–10.6)	< 0.001	0.27
85-yrs						
Both sexes (157)	17.2 (11.1–23.0)	< 0.001	0.66	17.8 (11.6–23.7)	< 0.001	0.65
Men (57)	22.8 (11.1–33.3)	< 0.001	0.56	21.1 (9.7–31.3)	< 0.001	0.59
Women (100)	14.0 (6.9–20.8)	< 0.001	0.71	16.0 (8.5–23.1)	< 0.001	0.68

EWGSOP; The European Working Groupon Sarcopenia in Older People 2010 (EWGSOP1 )[1] and 2019 (EWGSOP2) [2] diagnostic criteria (Table 1 and Table 3)

^a^ Difference, Exact binomial sign test

^b^ Agreement, Cohen’s kappa

### Mortality and ADL dependence

The median follow-up time was 5.2 years (range, 4.1—6.3) in the 70-year-olds and 3.7 years (range, 2.9—4.3) in the 85-year-olds. Mortality rates were 3.3% (*n* = 29) in the 70-year-olds and 15.3% (*n* = 24) in the 85-year-olds (Table 2). Sarcopenia according to both EWGSOP1 and 2 with − 2.5 T-score cut-offs were associated with increased mortality in the entire sample (HR 2.8 and 2.4 respectively, *P* < 0.05), but not when applying the − 2.0 T-score cut-offs (Table 7). In the 70-year-olds the hazard-ratios were 7.4 for EWGSOP1 (*P* = .001) and 7.6 for EWGSOP2 (*P* = .006) when applying the − 2.5 T-score cut-offs. Sarcopenia and severe sarcopenia were not associated with mortality in the 85-year-olds, regardless of diagnostic criteria (HR 1.2 to 3.2, *P* > 0.05) (see Table S4, Additional file 1).

**Table 7:** Associations between adverse outcomes and sarcopenia critera in the total sample of 70 and 85 year-olds

Sarcopenia criteria	All-cause mortality		ADL dependence^a^	
	HR (95%CI)^b^	*P*	OR (95%CI)^b^	*P*
Individual criteria (cut-off)^c^				
Hand grip strength (T-score − 2.0)	1.9 (1.0–3.9)	0.064	1.4 (0.9–2.1)	0.09
Hand grip strength (T-score − 2.5)	1.6 (0.7–3.3)	0.24	1.5 (1.0–2.4)	0.08
Muscle mass (T-score − 2.0)	1.4 (0.7–2.6)	0.34	1.2 (0.8–1.8)	0.44
Muscle mass (T-score − 2.5)	1.8 (0.9–3.7)	0.08	1.5 (0.9–2.5)	0.09
Muscle mass (EWGSOP1)^c^	1.4 (0.7–2.6)	0.32	1.2 (0.8–1.9)	0.35
Muscle mass (EWGSOP2)^c^	1.7 (0.9–3.2)	0.13	1.2 (0.8–1.9)	0.45
Gait speed (T-score − 2.0)	2.1 (1.1–4.0)	0.026	3.9 (2.6–6.0)	< 0.001
Gait speed (T-score − 2.5)	2.3 (1.2–4.6)	0.017	6.0 (3.6–10.0)	< 0.001
Gait speed (< 0.8 m/s)	2.5 (1.0–6.2)	0.042	9.3 (3.9–21.9)	< 0.001
EWGSOP (cut-off)				
1 (T-score − 2.0)	1.6 (0.8–3.3)	0.18	1.5 (0.9–2.4)	0.10
1 (T-score − 2.5)	2.8 (1.3–6.0)	0.009	2.2 (1.2–4.1)	0.012
2 (T-score − 2.0)	1.4 (0.7–2.9)	0.37	1.4 (0.8–2.3)	0.19
2 (T-score − 2.5)	2.4 (1.1–5.1)	0.029	1.8 (1.0–3.5)	0.062
1 (original)^b^	2.3 (1.0–4.9)	0.039	2.2 (1.2–3.9)	0.008
2 (original)^b^	2.4 (1.1–5.2)	0.028	2.2 (1.2–4.0)	0.007
1 & 2, severe (T-score − 2.0)	1.9 (0.9–4.1)	0.10	2.3 (1.2–4.4)	0.014
1 & 2, severe (T-score − 2.5)	2.0 (0.8–4.8)	0.12	3.6 (1.6–8.0)	0.002
1, severe (original)^c^	3.4 (1.0–11.4)	0.047	5.5 (1.2–24.2)	0.025
2, severe (original)^c^	3.4 (1.0–11.4)	0.047	5.5 (1.2–24.2)	0.025

^a^Barthel index < 100, *ADL* activities of daily living

^b^*HR* Hazard-ratio, *OR* Odds-ratio, *95%* confidence interval, adjusted for cohort and sex

^c^Diagnostic criteria and cut-offs as published by The European Working Groupon Sarcopenia in Older People 1 and 2 [1, 2] and population-based T-scores (Table 3)

Sarcopenia according to EWGSOP1 (− 2.5 T-scores), EWGSOP1 and 2 with “original” cut-offs and severe sarcopenia were associated with ADL dependence (*P* < 0.05) (Table 7). Gait speed was the only individual criteria associated with increased mortality and ADL dependence in the whole sample (Table 7). Stratified by cohort, gait speed was associated with ADL dependence in both cohorts, but with mortality only in the 70-year-olds (at T-score − 2.0 and − 2.5) (see Table S4 and S5, Additional file 1).

## Discussion

We assessed the prevalence of sarcopenia estimated by the EWGSOP1 and 2 criteria applying T-score cut-offs at two levels in a population-based samples of 70-year-olds born in 1944 and 85-year-olds born in 1930. In this way we studied the impact of the difference in operational definitions as well as the impact of different cut-off points on the prevalence of sarcopenia.

### EWGSOP 1 vs. 2

Overall, differences in sarcopenia prevalence were small and agreement was high between EWGSOP1 and EWGSOP 2, in both age groups, when applying the same cut-offs. However, in the 85-year-olds, the confidence interval of the difference in prevalence was wide, including a difference up to 8.2% at an overall prevalence of 42–62% (Table 5). Thus, a potential clinically meaningful difference in the older cohort could not be ruled out.

The sarcopenia prevalence will always be higher according to EWGSOP1 than 2 when using uniform cut-offs. The difference between the two operational definitions is the proportion of subjects with low performance but normal muscle strength (Table 1). Only three previous studies have compared EWGSOP 1 and EWGSOP 2 using uniform cut-offs (i.e., comparing operational definitions of sarcopenia) [8–10]. Locquet and colleagues reported a prevalence with EWGSOP1 and 2 of 11.6 and 7.4% respectively when applying the EWGSOP2 cut-offs (non-corrected ALSTI < 6.0 in women) in community-dwelling older adults (mean age 74 yrs.) [8]. Similar to us, Yang and colleagues found a slightly smaller difference applying population specific cut-offs in community-dwelling older adults (mean age 72 yrs.), 11.7 vs. 9.9% for EWGSOP1 and 2 respectively [9]. According to our study and these two studies, the EWGSOP2 operational definition decreases the prevalence by 0.8–4.2 percentage points when the overall prevalence is around 10%. However, Shafiee and colleagues found an unexpectedly poor agreement (Cohen’s kappa 0.34) and a large difference in prevalence, 7.9% (16.7 vs. 8.8%), between EWGSOP1 and 2, when applying uniform regional cut-offs in a large Iranian sample (mean age 69). This difference could be both method and population dependent which may need further exploration.

In the EWGSOP2, the cut-offs for low hand grip strength have been reduced for both sexes and for muscle mass in men compared to those suggested in EWGSOP1 [1, 2]. This should result in lower prevalence of sarcopenia, especially in men, that are separate from changes in operational definition. Consequently, most studies report that more individuals are classified as sarcopenic according to EWGSOP1 than according to EWGSOP2 with a difference around 2–6% (range − 2 to 20%) [8, 9, 11–14, 16–20, 34, 35]. Most studies have also found limited agreement between EWGSOP1 and 2 [8, 10, 14–21], while others have found a fair to good agreement [9, 11–13]. We found a substantial to almost perfect agreement between the two diagnostic criteria, especially when applying uniform T-score cut-offs.

### Prevalence

We found that the prevalence of sarcopenia was 1.4–7.8% in 70-year-olds and 42–62% in 85 years-old’s, depending on the criteria used.

In a cohort of Swedish community-dwelling men, mean age 86.6 yrs. [11], the prevalence with EWGSOP1 was 20% and with EWGSOP2 22%. In our study, the corresponding prevalence was 61 and 53% in 85-year-old men, respectively. Our high prevalence of sarcopenia in the 85-year-olds is also considerably higher than in most other studies within the similar age range, applying varying diagnostic criteria [36, 37]. The difference might be explained by the choice of method, cut-off, and reference population. We found a higher prevalence of low handgrip strength and low muscle mass than has been reported previously in the same age groups in Sweden and Denmark [11, 38]. The prevalence found in the 70-year-olds is within the range of other studies, though the range among studies is large [36, 37, 39].

### Impact of cut-off points

In the present study, a 0.5 T-score difference in cut-offs resulted in relatively large differences in the prevalence between these individual criteria, which translated to large differences in sarcopenia and severe sarcopenia prevalence (Tables 4, 6 and Table S3, Additional file 1). In the 70-year-olds a 5.8% difference (1.4 to 7.0%) and in the 85-year-olds a 17–18% difference (42 to 60%) in sarcopenia were observed. According to our results, the choice of cut-offs in a range suggested by the EWGSOP (i.e. -2.0 or − 2.5 T-scores) results in a difference in sarcopenia prevalence that is approximately 8 times larger than the difference due to the operational definitions of EWGSOP1 and 2 (Table 5, Table 6) [2].

### Mortality and ADL dependence

We found that sarcopenia and severe sarcopenia were associated with a more than two-fold higher risk of all-cause mortality in the entire sample of 70 and 85-year-olds when applying the − 2.5 T-scores or the “original” cut-offs (Table 7). In a meta-analysis of studies defining sarcopenia according to EWGSOP1, the pooled HR for all-cause mortality was 1.6 (range 1.25–3.89) [40]. In three studies that applied both EWGSOP1 and 2, HR estimates have ranged from 1.16 to 1.95 in adjusted models. Two of the three community-based studies found that EWGSOP1, but not 2, were significantly associated with mortality [16, 41, 42]. In our study no clear difference between the two diagnostic criteria can be established, however, due to overlapping HR estimates.

According to our survival analysis, sarcopenia at age 70 was associated with an increased relative risk of death, while at age 85, there was no significant association (see Table S4, Additional file 1). This might indicate that sarcopenia is a more serious condition at age 70. Since sarcopenia is closely related to ageing, it might be more normal being classified as sarcopenic at age 85 (i.e., primary sarcopenia) compared to at age 70. At this younger age, sarcopenia could be a sign of an underlying illness (i.e., secondary sarcopenia).

We found that gait speed was associated with both mortality and ADL dependence, whereas handgrip strength and muscle mass showed no associations. Reduced strength and slowness are well-known predictors of mortality and other adverse health outcomes [2, 43–46]. Low muscle mass is associated with ADL dependence, but its association with mortality in community-dwelling older adults is less clear [44, 47, 48]. The exclusion of gait speed as a criterion for sarcopenia in EWGSOP2 may reduce the prognostic abilities for adverse outcomes.

### Methodological considerations

There are some methodological challenges concerning cut-offs and testing procedures that need to be considered when interpreting and comparing results from different studies using the EWGSOP criteria. Firstly, in the EWGSOP1, specific cut-offs were not advised [1], but in the EWGSOP2, cut-offs were recommended to increase harmonization between studies. However, handgrip strength and gait speed are dependent on stature, and there are variations in muscle mass between populations in different geographical regions. Therefore, the use of regional normative population cut-offs (i.e., T-scores) is recommended [2, 49–51].

Secondly, how handgrip strength and gait speed are measured may also vary between studies (i.e., which hand, how many trials, a mean value for three trials or the highest value, walking distance and standing or rolling start) [9, 11]. Regarding gait speed, the cut-off suggested by EWGSOP, 0.8 m/s, is mainly derived from studies over short distances (2.4 to 6 m) and usually with a standing start [43, 46]. This procedure results in slower gait speed and reduced reliability compared to longer distances and a rolling start [52, 53]. For that reason, we derived cut-offs from Swedish studies using the same protocol as in our study (i.e., standing start over 30 m). In some studies, time up and go tests (TUG) are used instead of gait speed, which leads to a large difference in sarcopenia prevalence compared to using gait speed [17, 21, 50, 54]. Regarding handgrip strength, the type of device also affects results. The Jamar dynamometer is validated and widely used for measuring handgrip strength, but other devices, such as the Martin Vigorimeter, are also used [55]. Measures with the Jamar dynamometer and the Vigorimeter are highly correlated, show comparable reliability, and are probably clinically relevant to a similar extent [55–57]. However, the prevalence of low handgrip strength of 75–81% (at T-score − 2.5) in the 85-year-olds in the present study is considerably higher than the 42% found in a large British sample measured with the Jamar, from which the EWGSOP2 cut-offs (T-score − 2.5) were derived [58]. In the 70-year-olds, the prevalence was similar to the British sample (10–12% versus 9%) [58]. We speculate that the decline in strength at very old age might be assessed differently with a handle or a bulb dynamometer.

Thirdly, for assessment of muscle mass, several alternatives are available, and DXA is regarded as the most reliable for clinical purposes [59, 60]. Considerable differences among methods have been found, and there is limited agreement between different muscle mass adjustment techniques (i.e., height, BMI, etc.) [36, 61]. Taken together, these issues need consideration when comparing results from different studies.

According to our analysis, as well as others, differences in prevalence are mainly due to different cut-offs rather than the operational definition, and cut-offs, in turn, are dependent on different reference populations, measurement techniques, and procedures [17, 21, 36, 61].

These methodological variations hinder appropriate comparison between studies and populations and may have clinical implications leading to variations in treatment according to how sarcopenia is identified [36, 61].

### Study sample

As expected, given the 15-year age difference, there was a significant difference in sarcopenia prevalence between the 1944 and 1930 cohorts. However, for interpretation of results, it is important to consider that primary, as well as secondary non-response, was greater in the 1930 cohort compared to the 1944 cohort, meaning that the 85-year-olds probably is less representative for and in better health compared to the general population in this age group. We therefore suspect that the difference in sarcopenia prevalence between 70- and 85-year-olds is even greater in the general population. Differences between participants and non-participants were found within both age groups indicating that participants were healthier than the general population at the same age. Moreover, the 15-year age difference between cohorts does not only mean an effect of age but also period, and cohort effects should be considered [62], e.g., differences in living conditions during the life course. In the Gothenburg H70 Birth Cohort Studies, a positive trend has been shown through cohorts at the same age with later-born cohorts generally at better health [63–67].

### Strength and limitations

A strength in the present study is the use of a population-based and age-standardized sample. However, a considerable proportion of non-responders resulted in a small sample that may limit the generalizability of our findings, especially in the 85-year-olds and particularly among men of this cohort. Although our five and four-year follow-up of mortality is a v strength, the results should be interpreted with caution due to a small number of deaths during this timeframe. Comparing the birth cohorts, from a statistical point of view, the prevalence of sarcopenia, around 50% in 85-year-olds, gives stable values as opposed to the 70-yr-olds with a low prevalence, which leads to greater uncertainty in the true prevalence in the 70-year-olds. However, the smaller sample size of the 85-year-old cohort results in greater uncertainties in the estimates of prevalence and associations with mortality and ADL dependence. The use of DXA is a strength of this study [59, 60]. Handgrip strength was measured by the Martin Vigorimeter and gait speed was measured over 30 m, but no normalized reference cut-offs are present for these methods. We, therefore, constructed T-score cut-offs from a limited sample. On the one hand, this could be questioned and regarded as a limitation. On the other hand, the use of T-score cut-offs derived from a local population and with the exact same methods is a strength of this study. For this reason, the EWGSOP1 and 2 “original” were compared with the same cut-offs for handgrip strength, which is expected to make the difference between them smaller than when using the non-uniform published cut-offs [1, 2].

## Conclusion

In this cross-sectional study we found that the prevalence of sarcopenia was significantly higher in the 85-year-olds compared to 70-year-olds, irrespective of diagnostic criteria. Overall, the differences between the EWGSOP1 and 2 classifications were small within the same age group. The prevalence of sarcopenia was more dependent on cut-offs than on the operational definition. Meaningful differences in sarcopenia prevalence between EWGSOP1 and 2 in the 85-year-olds could not be ruled out. More research is needed to fully understand the predictive value of sarcopenia defined by EWGSOP1 and 2 in relation to morbidity and mortality. With this study, we hope to contribute to the emerging data on EWGSOP2 diagnostic criteria of sarcopenia and to the understanding of strategies leading to the implementation of standard measurements of sarcopenia in older adults.

## Supplementary Information

**Additional file 1 **: **Table S1**. Characteristics of participants and non-participants, **Table S2**. Difference in sarcopenia prevalence between EWGSOP1 and 2 using cut-offs as published by EWGSOP [1, 2], **Table S3**. Difference in the prevalence of severe sarcopenia when applying T-scores cut-offs at − 2.0 vs. -2.5, **Table S4.** Associations between mortality and sarcopenia critera stratified by cohort, **Table S5.** Associations between ADL dependence and sarcopenia critera stratified by cohort.

## Data Availability

An anonymized dataset is available to collaborative partners of the H70 Birth Cohort Studies study group after approved request. The senior author, Ingmar Skoog, can be contacted for further information (ingmar.skoog@neuro.gu.se).

## Abbreviations

ADL: Activities of daily living
ALSTI: Appendicular lean soft tissue index
BMI: Body mass index
DXA: Dual-energy X-ray absorptiometry
EWGSOP: European working group on sarcopenia in older people
H70: Gothenburg H70 Birth Cohort Studies
HR: Hazard ratio
OR: Odds ratio
TUG: Timed up and go
Yrs.: Years
WHO: World Health Organization
ICD: International Classification of Disease
