# Supplementary material for: Comparison of the 2010 and 2019 diagnostic criteria for sarcopenia by the European Working Group on Sarcopenia in Older People (EWGSOP) in two cohorts of Swedish older adults
Source: BMC Geriatr. 2021 Oct 26;21:600. doi: 10.1186/s12877-021-02533-y (PMC8547086; doi:10.1186/s12877-021-02533-y)
Supplement: Supplementary file 1 — Additional file 1 : Table S1. Characteristics of participants and non-participants, Table S2. Difference in sarcopenia prevalence between EWGSOP1 and 2 using cut-offs as published by EWGSOP [1, 2], Table S3. Difference in the prevalence of severe sarcopenia when applying T-scores cut-offs at − 2.0 vs. -2.5, Table S4. Associations between mortality and sarcopenia critera stratified by cohort, Table S5. Associations between ADL dependence and sarcopenia critera stratified by cohort. [file 12877_2021_2533_MOESM1_ESM.docx]

# Additional file 1

**Table S1**. Characteristics of participants and non-participants.

| Cohort |  | 70-year-olds  Born 1944 | |  | 85-year-olds  Born 1930 | |  |
| --- | --- | --- | --- | --- | --- | --- | --- |
|  |  | Non-participants | Included |  | Non-participants | Included |  |
|  |  | Count  (column %) | Count  (column %) | Difference  *P*-value^a^ | Count  (column %) | Count  (column %) | Difference  *P*-value^a^ |
| Sex | Man | 168 (53) | 391 (44) | 0.01 | 118 (35) | 57 (36) | 0.82 |
|  | Woman | 151 (47) | 493 (56) |  | 217 (65) | 100 (64) |  |
| Smoker | Never | 93 (30) | 358 (41) | <0.001 | 177 (55) | 75 (48) | 0.12 |
|  | Former | 170 (55) | 458 (52) |  | 130 (40) | 78 (50) |  |
|  | Current | 46 (15) | 66 (7) |  | 15 (5) | 4 (3) |  |
| Self-rated health | Very good | 95 (31) | 337 (38) | 0.002 | 55 (19) | 38 (24) | 0.03 |
|  | Fair to good | 179 (58) | 487 (55) |  | 177 (62) | 101 (64) |  |
|  | Decent to bad | 30 (10) | 58 (7) |  | 43 (15) | 18 (11) |  |
|  | Very bad | 5 (2) | 2 (0) |  | 12 (4) | 0 (0) |  |
| Education | Primary | 77 (24) | 106 (12) | <0.001 | 158 (49) | 69 (45) | 0.47 |
|  | Secondary or more | 241 (76) | 778 (88) |  | 165 (51) | 83 (55) |  |
| Living alone | Yes | 126 (40) | 315 (36) | 0.15 | 230 (69) | 101 (64) | 0.26 |
|  | No | 187 (60) | 568 (64) |  | 101 (31) | 56 (36) |  |
| Deceased^b^ | Alive | 286 (90) | 854 (97) | <0.001 | 213 (64) | 133 (85) | <0.001 |
|  | Dead | 32 (10) | 30 (3) |  | 122 (36) | 24 (15) |  |
|  |  |  |  |  |  |  |  |
|  |  | Mean±SD | Mean±SD |  | Mean±SD | Mean±SD |  |
| Medications (n) |  | 4.4±3.8 | 4±3.4 | 0.06 | 7.5±5 | 5.6±3.9 | <0.001 |
| BMI (kg/m2) |  | 27.1±5.1 | 25.7±4.3 | <0.001 | 25.3±4.6 | 25.3±3.9 | 0.97 |
| Arm circumference (cm) | Men | 30.2±3.9 | 30.2±3.3 | 0.81 | 30.9±23.9 | 28.8±3.4 | 0.50 |
|  | Women | 29.6±3.8 | 28.8±3.5 | 0.03 | 27.9±5.6 | 27.7±3.6 | 0.74 |
| Appendicular lean soft tissue (kg/m^2^) | Men | 7.8±1.1 | 7.9±0.8 | 0.72 | 6.6±0.8 | 6.8±0.8 | 0.56 |
|  | Women | 6.4±1.1 | 6.2±0.6 | 0.31 | 5.4±0.6 | 5.4±0.6 | 0.88 |
| Hand grip strength (kPa) | Men | 84±18 | 87±15 | 0.11 | 53±18 | 58±11 | 0.051 |
|  | Women | 73±14 | 74±14 | 0.51 | 49±15 | 49±14 | 0.96 |
| Gait speed 30m (m/s) | Men | 1.26±0.17 | 1.31±0.18 | 0.004 | 1.06±0.22 | 1.09±0.19 | 0.40 |
|  | Women | 1.26±0.20 | 1.30±0.20 | 0.014 | 1.04±0.24 | 1.02±0.19 | 0.77 |
|  |  |  |  |  |  |  |  |

^a^ Difference between included subjects, with complete data for sarcopenia measurements, and non-participants with missing data (proportions by Pearson Chi-2 and means by t-test).

^b^ Deceased as of 2020-25-06, log-rank test.

**Table S2**. Difference in sarcopenia prevalence (percentage points, %) between EWGSOP1 and 2 using cut-offs as published by EWGSOP [1, 2].

|  | Sarcopenia (cut-offs) |  |  |
| --- | --- | --- | --- |
|  | EWGSOP  1 vs. 2 (original^a^) |  |  |
| Cohort/Sex (n) | Difference % (95%CI) | *P*-value^b^ | *Κ*^c^ |
|  |  |  |  |
| All (1041) | 0.3 (-0.5–1.1) | 0.61 | 0.92 |
|  |  |  |  |
| 70-yrs |  |  |  |
| Both sexes (884) | 0.2 (-0.5–1.0) | 0.69 | 0.88 |
| Men (391) | 1.0 (-0.3–2.6) | 0.13 | 0.87 |
| Women (493) | -0.4 (-1.5–0.5) | 0.50 | 0.90 |
|  |  |  |  |
| 85-yrs |  |  |  |
| Both sexes (157) | 0.6 (-3.3–4.5) | 1.0 | 0.88 |
| Men (57) | 8.8 (0.7–16.5) | 0.063 | 0.82 |
| Women (100) | -4.0 (-8.2–0.3) | 0.13 | 0.92 |
|  |  |  |  |

^a^ Cut-offs as published by the EWGSOP 1 and 2 for muscle mass and gait speed [1, 2], and population based T-scores (-2.5T) for grip strength (Table 3).
^b^ Difference, Exact binomial sign test.
^c^ Agreement, Cohen’s kappa
EWGSOP; The European Working Groupon Sarcopenia in Older People 2010 (EWGSOP1)[1] and 2019 (EWGSOP2) [2] diagnostic criteria (Table 1).

**Table S3**. Difference in the prevalence of severe sarcopenia (percentage points, %) when applying T-scores cut-offs at -2.0 vs. -2.5.

|  | Severe sarcopenia (cut-offs) |  |  |
| --- | --- | --- | --- |
|  | EWGSOP1 or 2  T-score -2.0 vs. -2.5^a^ |  |  |
| Cohort/Sex (n) | Difference % (95%CI) | *P*-value^b^ | *Κ*^c^ |
|  |  |  |  |
| All (1041) | 2.7 (1.7–3.8) | <0.001 | 0.68 |
|  |  |  |  |
| 70-yrs |  |  |  |
| Both sexes (884) | 1.0 (0.3–1.9) | 0.004 | 0.31 |
| Men (391) | 0.3 (-0.8–1.5) | 1.0 | 0.67 |
| Women (493) | 1.6 (0.5–3.2) | 0.008 | 0.20 |
|  |  |  |  |
| 85-yrs |  |  |  |
| Both sexes (157) | 12.1 (6.9–17.5) | <0.001 | 0.68 |
| Men (57) | 12.3 (3.0–22.4) | 0.016 | 0.60 |
| Women (100) | 12.0 (5.3–18.8) | <0.001 | 0.71 |
|  |  |  |  |

^a^ Population based T-scores (Table 3).
^b^ Difference, Exact binomial sign test.
^c^ Agreement, Cohen’s kappa
EWGSOP; The European Working Groupon Sarcopenia in Older People 2010 (EWGSOP1)[1] and 2019 (EWGSOP2) [2] diagnostic criteria (Table 1).

**Table S4.** Associations between mortality and sarcopenia critera stratified by cohort.

|  | 70-year-olds Born 1944 | | 85-year-olds Born 1930 | |
| --- | --- | --- | --- | --- |
| Sarcopenia criteria | All-cause mortality | | All-cause mortality | |
|  | HR (95%CI)^a^ | *P* | HR (95%CI)^a^ | *P* |
| Individual criteria (cut-off)^b^ |  |  |  |  |
| Hand grip strength (T-score -2.0) | 2.3 (1.1-5.0) | 0.036 | 1.3 (0.3-5.7) | 0.69 |
| Hand grip strength (T-score -2.5) | 2.0 (0.8-5.2) | 0.17 | 1.3 (0.5-3.4) | 0.65 |
| Muscle mass (T-score -2.0) | 1.6 (0.7-4.0) | 0.30 | 1.2 (0.8-1.8) | 0.44 |
| Muscle mass (T-score -2.5) | 2.7 (0.9-7.9) | 0.07 | 1.4 (0.6-3.3) | 0.40 |
| Muscle mass (EWGSOP1)^b^ | 1.8 (0.4-2.9) | 0.87 | 1.7 (0.7-4.4) | 0.24 |
| Muscle mass (EWGSOP2)^b^ | 2.0 (0.8-4.8) | 0.14 | 1.4 (0.6-3.4) | 0.44 |
| Gait speed (T-score -2.0) | 2.5 (1.0-6.2) | 0.042 | 1.7 (0.7-3.9) | 0.23 |
| Gait speed (T-score -2.5) | 3.9 (1.3-11.3) | 0.012 | 1.7 (0.8-3.9) | 0.17 |
| Gait speed (<0.8 m/s) | 3.3 (0.4-24.4) | 0.25 | 2.8 (0.9-6.1) | 0.10 |
|  |  |  |  |  |
| EWGSOP (cut-off) |  |  |  |  |
| 1 (T-score -2.0) | 2.4 (0.8-6.9) | 0.11 | 1.3 (0.6-3.0) | 0.56 |
| 1 (T-score -2.5) | 7.4 (2.2-24.5) | 0.001 | 1.9 (0.8-4.4) | 0.12 |
| 2 (T-score -2.0) | 2.1 (0.6-7.1) | 0.24 | 1.2 (0.5-2.7) | 0.70 |
| 2 (T-score -2.5) | 7.6 (1.8-32.2) | 0.006 | 1.8 (0.8-4.1) | 0.15 |
| 1 (original)^b^ | 2.4 (0.6-10.2) | 0.23 | 2.2 (0.9-5.4) | 0.08 |
| 2 (original)^b^ | 4.3 (1.3-14.3) | 0.017 | 1.8 (0.8-4.3) | 0.16 |
| 1 & 2, severe (T-score -2.0) | 4.0 (0.5-30.3) | 0.18 | 1.7 (0.7-3.8) | 0.21 |
| 1 & 2, severe (T-score -2.5) | 33.1 (4.4-252) | 0.001 | 1.5 (0.6-3.9) | 0.36 |
| 1, severe (original)^b^ | - | - | 3.2 (1.0-10.8) | 0.059 |
| 2, severe (original)^b^ | - | - | 3.2 (1.0-10.8) | 0.059 |
|  |  |  |  |  |

^a^HR; Hazard-ratio, 95% confidence interval, adjusted for sex.
^b^Diagnostic criteria and cut-offs as published by The European Working Groupon Sarcopenia in Older People 1 and 2 [1, 2] and population-based T-scores (Table 3).

**Table S5.** Associations between ADL dependence and sarcopenia critera stratified by cohort.

|  | 70-year-olds Born 1944 | | 85-year-olds Born 1930 | |
| --- | --- | --- | --- | --- |
| Sarcopenia criteria | ADL dependence^a^ | | ADL dependence^a^ | |
|  | OR (95%CI)^b^ | P | OR (95%CI)^b^ | P |
| Individual criteria (cut-off)b |  |  |  |  |
| Hand grip strength (T-score -2.0) | 1.4 (1.0-2.2) | 0.08 | 1.3 (0.3-5.0) | 0.68 |
| Hand grip strength (T-score -2.5) | 1.6 (1.0-2.8) | 0.07 | 1.3 (0.5-3.1) | 0.59 |
| Muscle mass (T-score -2.0) | 1.1 (0.6-1.8) | 0.81 | 1.5 (0.7-3.4) | 0.32 |
| Muscle mass (T-score -2.5) | 1.1 (0.6-2.6) | 0.70 | 2.1 (1.0-4.6) | 0.059 |
| Muscle mass (EWGSOP1)^c^ | 0.8 (0.5-1.6) | 0.70 | 2.6 (1.1-6.0) | 0.03 |
| Muscle mass (EWGSOP2)^c^ | 0.9 (0.5-1.7) | 0.80 | 1.9 (0.9-4.4) | 0.11 |
| Gait speed (T-score -2.0) | 4.3 (2.7-7.1) | <0.001 | 2.8 (1.3-6.5) | 0.011 |
| Gait speed (T-score -2.5) | 7.5 (3.7-16.7) | <0.001 | 4.4 (2.0-9.4) | <0.001 |
| Gait speed (<0.8 m/s) | 23.7 (4.8-116) | <0.001 | 5.4 (1.8-15.7) | 0.002 |
|  |  |  |  |  |
| EWGSOP (cut-off) |  |  |  |  |
| 1 (T-score -2.0) | 1.5 (0.8-2.8) | 0.19 | 1.5 (0.7-3.2) | 0.34 |
| 1 (T-score -2.5) | 1.8 (0.6-5.7) | 0.30 | 2.3 (1.1-4.9) | 0.032 |
| 2 (T-score -2.0) | 1.4 (0.7-2.7) | 0.32 | 1.4 (0.6-3.0) | 0.40 |
| 2 (T-score -2.5) | 1.2(0.3-5.4) | 0.85 | 2.0 (1.0-4.3) | 0.065 |
| 1 (original)^c^ | 1.8 (0.7-4.7) | 0.20 | 2.6 (1.2-5.8) | 0.016 |
| 2 (original)^c^ | 2.5 (1.0-6.0) | 0.049 | 2.1 (1.0-4.5) | 0.060 |
| 1 & 2, severe (T-score -2.0) | 3.2 (0.9-11.4) | 0.062 | 1.9 (0.9-4.2) | 0.09 |
| 1 & 2, severe (T-score -2.5) | - | - | 4.0 (1.7-9.3) | 0.002 |
| 1, severe (original)^c^ | - | - | 5.4 (1.2-24.3) | 0.028 |
| 2, severe (original)^c^ | - | - | 5.4 (1.2-24.2) | 0.028 |
|  |  |  |  |  |

^a^Barthel index <100, ADL; activities of daily living ^b^OR; Odds-ratio, 95% confidence interval, adjusted for cohort and sex.
^c^Diagnostic criteria and cut-offs as published by The European Working Groupon Sarcopenia in Older People 1 and 2 [1, 2] and population-based T-scores (Table 3).
